# The Body Esteem Program: inspiring freedom from an eating disorder

**DOI:** 10.1186/2050-2974-2-S1-O62

**Published:** 2014-11-24

**Authors:** Kathleen Chinn, Jemma Caswell

**Affiliations:** 1 The Body Esteem Program, Perth, Australia

## 

WOMENS Healthworks developed the Body Esteem program (BEP) in 2005 to respond to consumer need for accessible and community-based services for women living with an eating disorder.

Core services include:

-20 week facilitated self-help groups for women over the age of 18 with Anorexia/Bulimia Nervosa or Binge Eating Disorder

- An Education and Support Workshop for family members or loved ones

- Positive Body Image presentations, projects and events.

- A Referral Directory

The unique aspect of the BEP is that all facilitators have lived experience and can bring their personal stories, recovery strategies and a sense of hope to the participants. Evaluations report that participants of the BEP feel less ashamed, less isolated and more confident and hopeful about the future. They are supported to set their own goals, make changes at their own pace and take responsibility for their own recovery process.

Viewed as a compliment to other treatment approaches, and funded by the WA Mental Health Commission, the BEP is involved in eating disorder sector development, regularly working alongside clinicians and services to provide valuable holistic support for recovery. Recently the BEP was invited to collaborate with Princess Margaret Hospital in order to develop, implement and evaluate eating disorder specific peer mentoring groups.

The BEP staff would like to share our program outcomes, ethos and management strategies at the 2014 ANZAED Conference.

This abstract was presented in the **Peer Support** stream of the 2014 ANZAED Conference.

